# Natural Killer Cell Deficiency in Neuroblastoma Amplified Sequence Gene Mutation

**DOI:** 10.7759/cureus.19270

**Published:** 2021-11-05

**Authors:** Rawia F Albar, Enad F Alsulimani, Khalid A Alsalmi, Abdulrahman Alnamlah, Abdullah Alhuzali, Saif Aljehani

**Affiliations:** 1 Pediatrics, King Abdulaziz Medical City, Jeddah, SAU; 2 Pediatrics, King Saud Bin Abdulaziz University for Health Sciences College of Medicine, Jeddah, SAU

**Keywords:** recurrent infection, interferon beta, neuroblastoma amplified sequence gene mutation, infantile liver failure syndrome, natural killer cell deficiency

## Abstract

Natural killer cell deficiency (NKD) occurs when decreased levels of such cells lead to major immunological deficiency in the patient. NK cells participate in tumor cell surveillance, viral infections, and immunoregulation in the body. We report a case of a nine-year-old female child, a known case of neuroblastoma amplified sequence (NBAS) gene mutation in the variant c.2819A>C (p. His940Pro), which causes infantile liver failure syndrome type 2 (ILFS2). The patient had been treated at four years of age for a three-day history of vesicular skin rashes in the L2 dermatome of the left leg, with pain and without swelling or redness, ear discharge, low appetite, and decreased activity. Also, she had already had multiple admissions due to different types of infections like viral hepatitis, urinary tract infection, Salmonella bacteremia, gastroenteritis, recurrent hepatitis, follicular tonsilitis, pneumonia, mastoiditis, and varicella-zoster infection. Flow cytometry revealed low levels of CD56^+^ and CD16^+ ^(2%). Recently, she has shown improvement by gaining weight and appetite following interferon-beta 1a injection.

## Introduction

Neuroblastoma amplified sequence (NBAS) gene is composed of 52 exons and is mapped to chromosome 2p24.3. It is highly present in cells of different tissues such as the brain, eye, hematopoietic cells, and connective tissue [1]. NBAS gene, a component of the soluble N-ethylmaleimide sensitive factor (NSF) attachment protein receptors, has a vital role in the Golgi-to-endoplasmic reticulum retrograde transport system. A decrease in the levels of the NBAS gene in the patients’ cells is accompanied by a reduction in p31, which is important for NBAS function in the soluble NSF attachment protein receptor complex [2].

Infantile liver failure type 2 or NBAS gene mutation is a rare autosomal recessive disorder that is characterized by a wide range of clinical symptoms including liver disease, growth retardation, skeletal and nervous system defect, immunological symptoms, frequent infections, hypogammaglobulinemia, and neutropenia. Most patients with NBAS-associated disease show all or some of these pathologies [3]. NBAS gene mutation was first observed in a Russian study to be associated with short stature, optic nerve atrophy, and Pelger-Huet anomaly (SOPH syndrome), but without liver failure [4]. Subsequent studies have found that the phenotypic spectrum of NBAS-based diseases also involves brain tissue, connective tissue, and the immune system [5].

Natural killer deficiency (NKD) is defined as a consistent and significant reduction in the numerical presence of NK cells, and results from inborn errors of the immune system that hamper the development of NK cells, impair the function thereof, or both. NKD can be broadly classified into four subcategories: (1) isolated NKD due to a genetic defect; (2) isolated NKD that is neither associated with a genetic defect nor a known pathology; (3) NKD associated with a disease that is the result of a known genetic defect that secondarily causes an immune deficiency; and (4) NKD associated with a disease that is not due to a genetic etiology [6].

Two case reports of complete deficiency of NK cells in humans have described the importance of NK cells in the protection against infections and immune surveillance. In the first case, the patient suffered from recurrent herpes virus infections and multiple bacterial pathogens [7]; the other case suffered from recurrent anogenital warts and valvular and cervical cancer caused by human papillomavirus (HPV) [8].

In this report, we present a case of NBAS gene mutation and episodic liver failure associated with NKD and recurrent infections, focusing on its immunological management.

## Case presentation

The patient was a nine-year-old female, a known case of NBAS gene mutation in the variant c.2819A>C (p. His940Pro), which causes infantile liver failure syndrome type 2 (ILFS2) (Table 1). At the age of four years, she had been brought with a three-day history of vesicular skin rashes in the L2 dermatome, of the left leg, with pain and without swelling or redness, low appetite, and ear discharge. The patient is the daughter of consanguineous parents, born of a full-term pregnancy, and had an unremarkable neonatal period. She had been admitted as a case of varicella-zoster and methicillin-resistant Staphylococcus aureus (MRSA) ear infection and managed by acyclovir IV (15 mg/kg) for 10 days, and vancomycin IV (15 mg/kg) for 14 days.

From the age of two months to four years, she had been hospitalized several times due to multiple infections such as viral hepatitis, urinary tract infection, Salmonella bacteremia, gastroenteritis, recurrent hepatitis, follicular tonsilitis, pneumonia, mastoiditis, and varicella-zoster infection. Flow cytometry had been performed when she had been admitted due to varicella infection at the age of four years and had shown low levels of CD56+ and CD16+ (2%) but had otherwise been normal. Thus, the patient was diagnosed with NKD and was recommended to take 10 mcg interferon-beta 1a injections once per week, to which the patient responded very well.

After the initiation of interferon-beta 1a injections, the patient was doing very well for five months. However, despite receiving proper management, she developed mild upper respiratory tract infection and otitis media. The medical team advised increasing the dose of interferon-beta 1a to 15 mcg once per week as a precautionary measure to minimize the probability of future infections. However, it was suspected that if the patient stopped using the interferon injections, she would develop infections and flareups of her disease as had been the case when she had run out of interferon injections a year after she had been prescribed interferon. She was admitted to the ICU as a case of acute follicular tonsillitis and flareup of hepatitis, which were managed by antibiotics and increasing the dosage and frequency of the interferon-beta 1a injections to 22 mcg three times per week.

The patient currently is nine years old and on 22 mcg interferon-beta 1a injection three times per week. There has been a marked improvement in weight gain and appetite. Besides, the frequency of infections has remarkably decreased.

**Table 1:** Whole-exome sequencing Whole-exome sequencing identified the variant c.2819A>C (p. His940Pro) in the NBAS gene NBAS: neuroblastoma amplified sequence; OMIM: Online Mendelian Inheritance in Man; MAF: minor allele frequency

Gene (transcript)	Disorder (OMIM#, inheritance)	Nucleotide (protein)	Zygosity			Described by	MAF	In silico parameters	Variant classification
NBAS (NM_015909.3)	Infantile liver failure syndrome type 2 (616483, AR)	c.2819A>C (p. His940Pro)	Index	Mother	Father	Not described	Not reported	4/4: damaging	Uncertain significance (class 3)
			Hom.	Het.	Het.				

## Discussion

NK cells are lymphocytes of the innate immune system that play a major role in the immune response to viral infections and cancerous tumors by contact mitigation and the killing of target cells as well as cytokine secretions, both of which are done by a series of germline-encoded activating and inhibitory receptors [9]. They develop from the common lymphoid progenitor, comprise 5-15% of peripheral blood lymphocytes, and do not express T cells and immunoglobulin on their surface. NK cells are further classified into two subgroups: CD56bright and CD56dim, each of which possesses a unique phenotype and function. CD56bright is responsible for the production of cytokines, particularly IFN-γ. And CD56dim constitutes most NK cells in the blood and specializes in mediating target cell lysis [10].

NBAS gene mutation has been linked to immunodeficiencies in the literature. Ricci et al. examined a patient who exhibited symptoms since birth, who had been born via an emergency Cesarian section. The patient also experienced severe intrauterine growth restriction and blood flow abnormalities [11]. NKD patients are not necessarily diagnosed at a young age. For example, the patient investigated by Zhang et al. was diagnosed as an adult [6]. Our patient had been born full-term via spontaneous vaginal delivery to consanguineous parents, and her neonatal period had been generally unremarkable. She had experienced a high frequency of recurring infections, which prompted us to suspect immunodeficiencies in her. Both our patient and the one investigated by Ricci et al. were screened using whole-exome sequencing, but unlike our patient, who exhibited a mutation in the variant c.2819A>C, their patient exhibited a c.1948C>T variant mutation [11]. In the studies by Ricci et al. and Lenz et al., general symptoms of NBAS-linked immunodeficiencies were shown to be decreased levels of immunoglobulin G, decreased CD56+ NK cells and CD19+ naive B cells, and persistent infections [11,12]. In Ricci et al.'s study, the patient mainly exhibited a Klebsiella pneumoniae urinary tract infection and MRSA sepsis [11]. In Zhang et al.'s paper, the patient displayed normal IgG levels but was hepatitis B- and C-positive and had developed enlarged lymph nodes as a child [5]. Our patient experienced urinary tract infections and mainly throat infections (upper respiratory tract infections), including the varicella infection, which led the medical team to perform a flow cytometry test.

When treating NBAS-linked immunodeficiencies, immunoglobulin substitutive therapy appears to be the dominant method along with the use of corticosteroid and antimicrobial agents as needed. Ricci et al. also treated their patient with antimicrobial prophylaxis and corticosteroid therapy, which resulted in the almost immediate reduction of peripheral hypereosinophilia. Steroid tapering, however, was unsuccessful and caused the re-exacerbation of the patient’s gastrointestinal symptoms. Our patient, on the other hand, was mainly administered interferon-beta whenever she presented to the hospital with an infection. Her prognosis at the moment seems to be highly favorable, as the frequency of her infections has decreased, and her weight has increased [11].

The management plan and therapeutic treatment in patients presenting with NKD are diverse and vary from one case to another. However, most of the studies have focused on managing herpesviruses and other opportunistic infections [7,13]. Acyclovir is an antiviral medication that has been prescribed in anecdotal cases. Other ointment agents and immunomodulators have been used in cases of HPV infections. Due to the high risk of being susceptible to HPV, the HPV vaccine should be given to almost all patients diagnosed with NKD [14].

## Conclusions

Defective NBAS is a rare disorder with an unknown incidence rate. Its clinical manifestations include repeated liver damage and optic atrophy. Currently, the clinical diagnosis of the disease is challenging, but there are many treatment modalities. We recommend that the clinicians start early immunotherapy in appropriate doses for NKD patients to prevent recurrence of infection.
